# Effectiveness estimates of three COVID-19 vaccines based on observational data from Puerto Rico

**DOI:** 10.1016/j.lana.2022.100212

**Published:** 2022-02-24

**Authors:** Mónica M. Robles-Fontán, Elvis G. Nieves, Iris Cardona-Gerena, Rafael A. Irizarry

**Affiliations:** a CDC Foundation-Puerto Rico Department of Health, Río Piedras, PR, United States; b Puerto Rico Department of Health, Río Piedras, PR, United States; c Department of Data Science, Dana-Farber Cancer Institute, CLSB 11007, 450 Brookline Ave, Boston, MA 02215, United States; Department of Biostatistics, Harvard T.H. Chan School of Public Health, 677 Huntington Ave, Boston, MA 02115

**Keywords:** SARS-CoV-2, COVID-19, Vaccines, Observational study

## Abstract

**Background:**

On July 15, 2021, with 58% of the population fully vaccinated, the start of a COVID-19 surge was observed in Puerto Rico. On July 22, 2021, the government of Puerto Rico started imposing a series of strict vaccine mandates. Two months later, over 70% of the population was vaccinated, more than in any US state, and laboratory-confirmed SARS-CoV-2 had dropped substantially. The decision to impose mandates, as well as current Department of Health recommendations related to boosters, were guided by the data and the effectiveness estimates presented here.

**Methods:**

Between December 15, 2020, when the vaccination process began in Puerto Rico, and October 15, 2021, 2,276,966 individuals were fully vaccinated against COVID-19. During this period 112,726 laboratory-confirmed SARS-CoV-2 infections were reported. These data permitted us to quantify the outcomes of the immunization campaign and to compare effectiveness of the mRNA-1273 (Moderna), BNT162b2 (Pfizer), and Ad26.COV2.S (J&J) vaccines. We obtained vaccination status, SARS-CoV-2 test results, and COVID-19 hospitalizations and deaths, from the Department of Health. We fit statistical models that adjusted for time-varying incidence rates and age group to estimate vaccine effectiveness, since the time of vaccination, against lab-confirmed SARS-CoV-2 infection, and COVID-19 hospitalization and death.

**Results:**

Two weeks after final dose, the mRNA-1273, BNT162b2, and Ad26.COV2.S vaccines had an effectiveness of 90% (95% CI: 88–91), 87% (85–88), and, 64% (58–69), respectively. After five months, effectiveness waned to about 70%, 50%, and 40%, respectively. We found no evidence that effectiveness was different after the Delta variant became dominant. For those infected, the vaccines provided further protection against COVID-19 hospitalization and deaths across all age groups, and this conditional effect did not wane in time.

**Interpretation:**

The mRNA-1273 and BNT162b2 vaccines were highly effective across all age groups. They were still effective after five months although the protection against SARS-CoV-2 infection waned. The Ad26.COV2.S vaccine was effective but to a lesser degree compared to the mRNA vaccines. Although, conditional on infection, protection against adverse outcomes did not wane, the waning in effectiveness resulted in a decreased protection against serious COVID-19 outcomes across time.

**Funding:**

RAI's work was partly funded by NIH Grant R35GM131802.

**Research in context**

**Evidence before this study**

Two mRNA COVID-19 vaccines and one viral vector COVID-19 vaccine were proven to be safe and effective in randomized clinical trials during late 2020 and early 2021, respectively. The effectiveness of these vaccines on SARS-CoV-2 outcomes has been stated in several observational studies with different settings such as healthcare workers, integrated health care systems, etc. Most studies compared incidence rates between the vaccinated group and the unvaccinated group. A vaccinated group comprehended people who had completed a vaccination series for at least 7 days while the unvaccinated group inclusion criteria were more variable going from people with no vaccine dose, one dose with more than 7 days after the administration date, and less than 7 days after vaccination series is completed. The research published usually comprises the assessment of one or two vaccines against one or more SARS-CoV-2 outcomes. The most comprehensive study in terms of SARS-CoV-2 outcomes assessed one mRNA vaccine, while studies that investigate more than one vaccine have excluded parts of the population.

**Added value of this study**

This study provides a complete overview of vaccination efforts and vaccine effectiveness against SARS-CoV-2 outcomes in a real-world scenario for a unique population in a certain period. For all three vaccines, effectiveness is measured for three SARS-CoV-2 outcomes under equal conditions. This provides a unique opportunity to assess and appropriately compare vaccine performance and how it changes as time passes since the final dose. Furthermore, in this research, it is shown that vaccine effectiveness against SARS-CoV-2 infections remained seemingly unaffected during the dominance of the Delta variant of concern.

**Implications of all the available evidence**

The success of public health interventions relies heavily on accurate scientific results that reflect the real world. This research has already had implications in public health interventions in Puerto Rico, as an earlier version provided a scientific base for recommendations related to boosters guided by our time-varying effectiveness estimates. The work was also shared by the Puerto Rico Department of Health with the Centers of Disease Control and the US White House COVID-19 Task Force to help with the deliberations about the need for boosters. Furthermore, this research provides evidence that the effectiveness of all vaccines decreased over time, implying that to reach herd immunity boosters shots will be needed. Because our analysis was performed with completely reproducible computer code we will be able to update our results as new data comes in and continue to guide public health decisions.

Alt-text: Unlabelled box

## Introduction

The first COVID-19 case was first reported on December 31, 2019, and the race for a vaccine commenced shortly after. By fall 2020, there were several ongoing clinical trials for vaccines developed with full messenger RNA (mRNA) technology.^1^ In the United States, three of the currently used COVID-19 vaccines were shown to be highly effective against SARS-CoV-2 outcomes in randomized double-blind trials. A two-dose series of BNT162b2 (Pfizer) COVID-19 vaccine was shown to be 95% efficacious against symptomatic COVID-19 in individuals 16 and older.^2^ Similarly, a two-dose series of mRNA-1273 (Moderna) vaccine provided 94% efficacy against symptomatic COVID-19 for those 18 and older.^3^ Finally, a single-shot of the Ad26.COV2.S (Johnson & Johnson) COVID-19 vaccine provided 66% protection against moderate to severe COVID-19 disease for individuals 18 years and older.^4^ These findings led to emergency use authorizations (EUA) by the Federal Drug Administration (FDA) for these three vaccines. In August 2021, the BNT162b2 COVID-19 vaccine received FDA approval for the prevention of COVID-19 disease for individuals 16 years and older.^5^ As of October 15 2021, over 400 million doses of these vaccines have been administrated in the United States.

Since the start of the vaccination process, the effectiveness of the COVID-19 vaccines has been estimated in several observational studies. A population-based study performed early during the vaccination process in Israel estimated the BNT162b2 vaccine effectiveness against infection to be 66–85% and over 90% against COVID-19 hospitalization.^6^ A matched cohort study, also in Israel, found 92% effectiveness against SARS-CoV-2 infection, 87% effectiveness for hospitalizations, and 72% effectiveness at preventing COVID-19 death for the BNT162b2 vaccine.^7^ A study conducted in inpatient care settings, across 187 hospitals in the United States, found that the mRNA-1273, BNT162b2, and Ad26.COV2.S COVID-19 vaccines were highly effective against COVID-19 related hospitalization, ICU admission, and emergency department or urgent care clinic visit.^8^ Other observational studies have estimated vaccine effectiveness amid variants of concern. The effectiveness against SARS-CoV-2 infection in the dominance of the Delta (B.1.617.2) variant has been shown to remain at 88% for the BNT162b2 COVID-19 vaccine.^9^ Similarly, a population-based observational study found that two doses of the mRNA-1273 and BNT162b2 COVID-19 vaccines provided substantial protection against the Alpha (B.1.1.7), Beta (B.1.351), Gamma (P.1), and Delta variants of concern.^10^ Nonetheless, real-world estimates comparing the mRNA-1273, BNT162b2, and Ad26.COV2.S COVID-19 vaccines effectiveness against SARS-CoV-2 infections, COVID-19 hospitalizations and deaths, that consider time since initial inoculation, on the same population, is still limited. Here we add to these findings by examining the effect of vaccines on all laboratory-confirmed SARS-CoV-2 infections observed in Puerto Rico since the vaccination process started, which provided enough power to estimate effectiveness across age groups and as a function of time since the individuals are considered fully vaccinated (two weeks after final dose).

Following the emergency approval of the BNT162b2 vaccine manufactured by Pfizer-BioNTech on December 11, 2020, vaccine administration began in Puerto Rico on December 15, 2020. The logistics of the vaccination process proposed by the Puerto Rico Department of Health established that the vaccine administration would be completed in phases, as recommended by the World Health Organization (WHO) and the Centers for Disease Control and Prevention (CDC).^11^ The phases (***Supplementary Table* S1**) consisted of vaccinating populations that were at greater risk of infection and severe disease. Front line health care workers and people 65 years and older were vaccinated in the first phase of the vaccination rollout.

By October 15, 2021 more than 2 million of the 3,285,874 individuals living in Puerto Rico had completed the COVID-19 vaccination series. Furthermore, since the start of the vaccination process until October 15, 2021, Puerto Rico experienced two SARS-CoV-2 infection surges, one starting in late March after restrictions were lifted and another one in late-June, with the arrival of the Delta variant. For this period, 112,726 SARS-CoV-2 infections have been detected. We leveraged data collected by the Puerto Rico Department of Health to study the effect of the vaccination process in preventing SARS-CoV-2 outcomes by comparing unvaccinated individuals 12 years or older to those who had completed the vaccination series (two weeks after final dose) for mRNA-1273, BNT162b2, or Ad26.COV2.S COVID-19 vaccines. We provide estimates of the effectiveness of the three COVID-19 vaccines protecting against SARS-CoV-2 infections, COVID-19 hospitalizations, and deaths to quantify the public health impact of the vaccine in Puerto Rico.

## Methods

### Data source and collection

Two Puerto Rico Department of Health databases were integrated: the BioPortal, which stores laboratory SARS-CoV-2 test results, most COVID-19 hospitalizations, and COVID-19 deaths, and the Puerto Rico Electronic Immunization System (PREIS), which stores vaccination related data, including COVID-19 vaccination. The Department of Health used full names (including maternal last names), date of birth, and municipalities to match the two databases. Because over 200 billion records needed to be compared, an algorithm was developed to do this automatically. Using a smaller subset curated by a human, we determined the algorithm to provide matches that well over 99% accurate. Note that the Veteran Health Administration, Department of Defense, and Federal Bureau of Prisons do not share individual level data with state public health agencies. Based on aggregate data shared by the CDC, we estimate that only about 1.6% of vaccine doses administered in Puerto Rico were administered by these entities.

### Statistical analyses

Here we describe the approach used to estimate vaccine effectiveness as a function of time since becoming fully vaccinated. Note that this is different from estimating effectiveness as a function of calendar date since the date individuals became fully vaccinated vary from January to October. Because the vaccination process started at different dates for the different age groups and Puerto Rico had two SARS-CoV-2 infections surges during the study period, we had to develop a statistical analysis that accounted for time-varying incidence rates and age. Below we provide a general description, with the mathematical details in the ***Supplementary Methods***.

### Estimating effectiveness since time of vaccination

We denoted an individual as fully vaccinated two weeks after the date they received the final dose in the COVID-19 vaccine series. SARS-CoV-2 infections in which the laboratory confirmation occurred after the first dose but before being fully vaccinated were removed from the analysis. For SARS-CoV-2 infections in which the laboratory confirmation occurred after being fully vaccinated, we recorded the number of days the individual had been fully vaccinated on the date their positive test was recorded. We denoted this value with d. Then for each date t and each demographic stratum (defined by age group and gender) we computed the number of laboratory-confirmed SARS-CoV-2 infections among those that had been vaccinated for d days. We denoted these counts with $Y_{t,d}$. To compute rates, we calculated, for each date t, the number of individuals in this stratum that became fully vaccinated d days before a date t. We denoted this population size with $N_{t,d}$.

To estimate the time varying relative risk as a function d, denoted with RR(d), we assumed that the counts $Y_{t,d}$ followed a Poisson distribution with rate $N_{t,d}\times \mu_{t}\times RR\left(d\right)$, with $\mu_{t}$ the rate for the unvaccinated on date t, and RR(d) a smooth function of d. Note that 1−RR(d) is equivalent to vaccine effectiveness.

To be able to estimate $\mu_{t}$ we computed the number of laboratory-confirmed SARS-CoV-2 infections among the unvaccinated in the same demographic group, and, to compute rates, we calculated, for each date t, the number of individuals in the stratum that were unvaccinated and had not been infected within the last three months. We combined these data, the $Y_{t,d}$ counts from all strata, and fit a generalized linear model to estimate RR(d), and point-wise confidence intervals for each vaccine manufacturer, using data from all strata. Details are included in the ***Supplementary Appendix***.

Note that this model can be fit to any demographic strata and any vaccine manufacturer to obtain vaccine effectiveness in these contexts. Furthermore, the approach can be applied to COVID-19 hospitalization or deaths by simply redefining $Y_{t,d}$ with these outcomes rather than laboratory-confirmed SARS-CoV-2 infections.

### Estimating risk of hospitalization and death since time of vaccination conditioned on a laboratory-confirmed SARS-CoV-2 infection

In this section, to avoid introducing more mathematical symbols, we re-purpose the Y and N notation.

To estimate the further protection provided by the vaccine in reducing adverse outcomes (COVID-19 hospitalizations or deaths) among infected individuals we examined the proportion of laboratory-confirmed SARS-CoV-2 infections that had COVID-19 hospitalizations or deaths. We counted all hospitalizations and deaths that met the Health and Human Services (HHS) criteria for COVID-19 for individual tested positive anytime previous to that determination.

For each date t and each demographic stratum we computed the number of laboratory-confirmed SARS-CoV-2 infections with an adverse outcome (COVID-19 hospitalization or death) among those that had been vaccinated for d day. We denoted these counts with $Y_{t,d}$. Note that now, instead of obtaining the population from all vaccinated individuals to compute rates, we only considered individuals with laboratory-confirmed SARS-CoV-2 infections. Therefore, for each date t, we defined $N_{t,d}$ as the number of individuals in this stratum with laboratory-confirmed SARS-CoV-2 infections among those that had been vaccinated for d day. We then assumed that $Y_{t,d}$ followed a binomial distribution with $N_{t,d}$ trials and success probability p(d) assumed to be a smooth function of d. The probability p(d) represents the time-varying risk of the adverse outcome conditional on infection. We compared this function to the conditional risk of unvaccinated individuals for each demographic group computed as the proportion of laboratory-confirmed unvaccinated individuals having adverse outcomes.

Note that this model can be fit to any demographic stratum and any vaccine manufacturer to obtain time-varying risks in these contexts.

### Role of the funding source

NIH Grant R35GM131802 partially funded the work by RAI to develop the statistical methods. The funders had no role in study design, data collection, data analysis, interpretation, or writing of the report.

## Results

### Vaccination data

**Figure 1:**
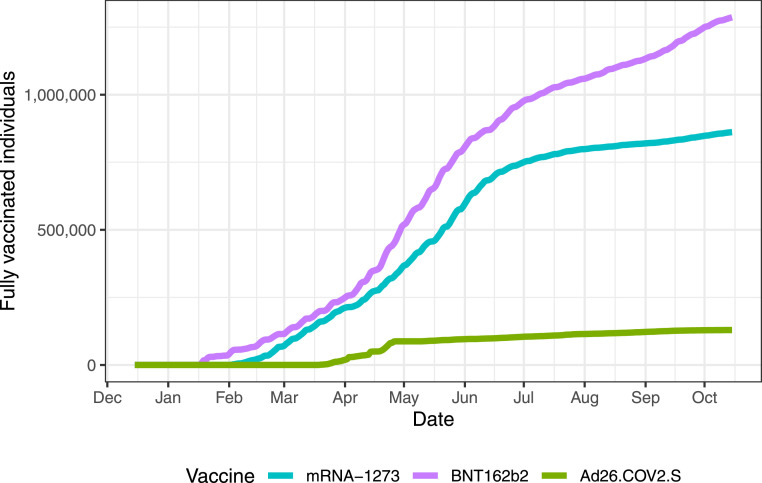
Cumulative number of fully vaccinated individuals per day by vaccine manufacturer (represented with different colors).

As of October 15, 2021, 2,276,966 of the 3,285,874 individuals living in Puerto Rico had been fully vaccinated: 1,286,293 with BNT162b2; 861,378 with mRNA-1273 and 129,295 with Ad26.COV2.S. The daily COVID-19 vaccination rates were high during the first four months of the vaccination process, successfully vaccinating 50% of the eligible population (1,442,459 of 2,848,293) with at least one dose of a COVID-19 vaccine.^12^ However, daily vaccination rates decreased after reaching 50%. The decrease in daily rate was particularly noticeable among the older age groups. After several vaccine mandates were implemented in July and August, the rate started to increase again (Figure 1) and by October 17, 2020, over 70% of the population was fully vaccinated - more than in any US state. With the exception of pediatric population (12–17 year-old children) who were only vaccinated with BNT162b2, the age distribution of the vaccine was similar for all vaccine manufacturers (***Supplementary Figure* S1**). However, the age distribution of the vaccinated population was different before and after Delta became dominant (***Supplementary Table* S2**).

### Observed SARS-CoV-2 infections, COVID-19 hospitalizations, and deaths

**Figure 2:**
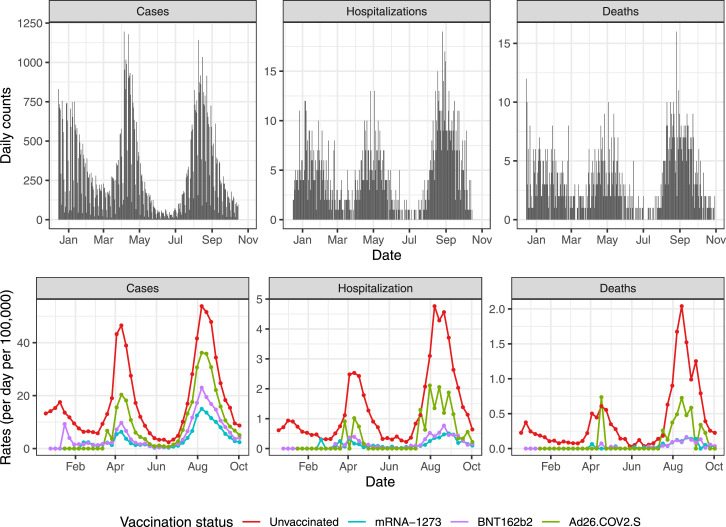
(Top): SARS-CoV-2 infections (Cases), COVID-19 hospitalizations and deaths per day for individuals 12 years or older during the period of study. (Bottom): Weekly rates (per day per 100,000) for SARS-CoV-2 infections (Cases), COVID-19 hospitalizations and deaths by vaccination status. The rates are based on total numbers and are not adjusted for age. Colors are used to denote the different vaccination status.

During the period considered in this analysis (December 15, 2020 to October 15, 2021), 88,704 laboratory-confirmed SARS-CoV-2 infections were observed for individuals 12 years or older. These were mostly distributed across two surges (Figure 2). The first surge started shortly after several restrictions were lifted on March 15, 2021. Restrictions were imposed again on April 9, 2021, and the cases decreased to a level not seen since the summer of 2020. As a result, restrictions were lifted once again on May 24, 2021, and shortly after the arrival of the Delta variant, a second surge began by the end of June 2021.^13^ The SARS-CoV-2 infections observed during the period of December 15, 2020 to October 15, 2021 resulted in at least 5,028 and 1,371 COVID-19 hospitalizations and deaths, respectively (Figure 2; ***Supplementary Table* S3**).

Laboratory-confirmed SARS-CoV-2 infections, COVID-19 hospitalization, and COVID-19 death rates were noticeably lower among the fully vaccinated (***Supplementary Table* S3**). The protection afforded by the vaccine was observed by simply plotting the rates by vaccination group (Figure 2). The relative risk for the vaccinated seemed to increase after June 15, 2021, the period in which the Delta variant dominated. However, these rates are not adjusted for age and potential waning effectiveness was also not considered. Therefore, to better assess COVID-19 vaccine effectiveness, we applied the approach described above in the *Methods Section*.

### Time-varying vaccine effectiveness against laboratory-confirmed SARS-CoV-2 infections

We fit a statistical model to estimate time-varying vaccine effectiveness while accounting for the difference in demographics between vaccinated and unvaccinated individuals. To evaluate whether the Delta variant affected vaccine effectiveness, we considered two periods: before and after June 15, 2021. For this particular analysis, to ensure we had enough data in each stratum in both time periods (see ***Supplementary Figure* S2**), we considered a range of 0–90 days since fully vaccinated, combined the individuals over 65 into one age group, and excluded Ad26.COV2.S from the analysis. Although the Delta variant led to more SARS-CoV-2 infections (Figure 2A), a decrease in vaccine effectiveness was not detected, consistent with this variant being more contagious yet the vaccines equally effective against it. Note also that we did not find statistically significant differences among the age groups.

Considering that no clear differences were observed across age groups and time periods, we estimated one time-varying effectiveness across all age groups and for the entire vaccination process period. Because the period of evaluation was different for the different age groups, due to the previously described vaccine rollout phases (***Supplementary Table* S1**), we included length of time since vaccination that was possible for the entire population: 144 days for mRNA-1273, 151 for BNT162b2, and 172 for Ad26.COV2.S. Because vaccine administration for individuals between 12 and 17 years only started on May 12, 2021, and was restricted to the BNT162b2 vaccine, we also excluded 12–17 year old individuals from this analysis.

**Figure 3:**
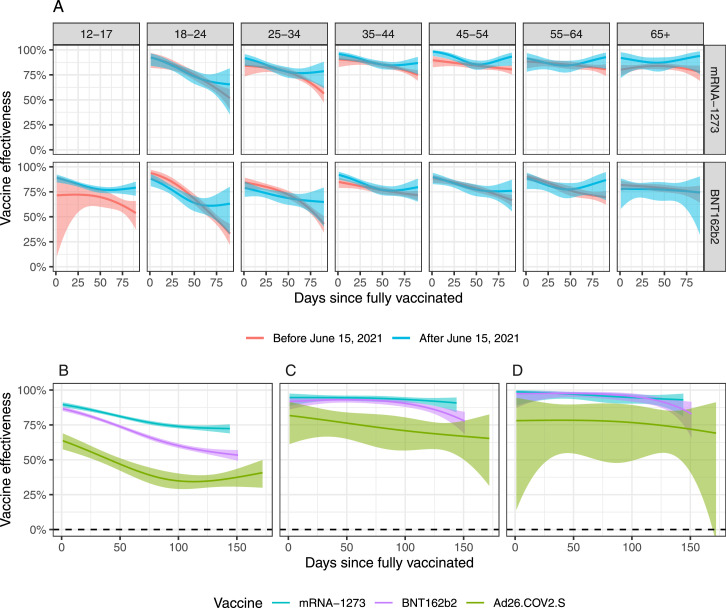
Estimated effectiveness plotted against days since the individuals were fully vaccinated. The ribbons represent point-wise 99% confidence intervals. (A) Comparison before and after the Delta variant became dominant by age group and vaccine manufacturer (mRNA-1273 and BNT162b2). (B) Vaccine effectiveness against infections by manufacturer for the entire study period and age groups combined. (C) same as B, but for hospitalizations. (D) same as B but for death.

The mRNA-1273, BNT162b2, and Ad26.COV2.S started out with vaccine effectiveness of 90% (95% CI: 88–91), 87% (85–88), and 64% (58–69), respectively. Effectiveness then started waning to 72% (69–75), 54% (51–57), and, 36% (31–42), respectively, at the end of the study period (Figure 3B Table 1).

**Table 1:** Waning effectiveness against infection with 99% point-wise confidence intervals.

Outcome	Vaccine	Effectiveness on first day as fully vaccinated (CI)	Effectiveness after 144 days (CI),
Infection	mRNA-1273	90% (88–91%)	72% (69–75%)
Infection	BNT162b2	87% (85–88%)	54% (51–57%)
Infection	Ad26.COV2.S	64% (58–69%)	36% (31–42%)
Hospitalization	mRNA-1273	95% (89–97%)	91% (84–95%)
Hospitalization	BNT162b2	92% (86–95%)	81% (74–86%)
Hospitalization	Ad26.COV2.S	82% (61–91%)	67% (54–77%)
Death	mRNA-1273	99% (89–100%)	93% (81–97%)
Death	BNT162b2	97% (87–99%)	86% (76–92%)
Death	Ad26.COV2.S	78% (14–94%)	73% (49–86%)

### Time-varying vaccine effectiveness against COVID-19 hospitalizations and deaths

We fit the same model to estimate relative risk of COVID-19 hospitalization and deaths. For hospitalizations we saw even higher effectiveness for mRNA-1273, BNT162b2, and Ad26.COV2.S started out with vaccines effectiveness of 95% (95% CI: 89–97), 92% (86–95), and 82% (61–91), respectively. Effectiveness then started waning to 91% (84–95), 81% (74–86), and 67% (54–42), respectively, after 144 days. For deaths, we saw even higher effectiveness: mRNA-1273, BNT162b2, and Ad26.COV2.S started out with vaccines effectiveness of 99% (95% CI: 89-100), 97% (87-99), and 78% (14–94), respectively (Figure 3). Effectiveness then commenced waning to 93% (81–97), 86% (76–92), and 73% (49–42), (Figure 3C and D Table 1).

### Vaccines lower the risk of COVID-19 hospitalization and death

**Figure 4:**
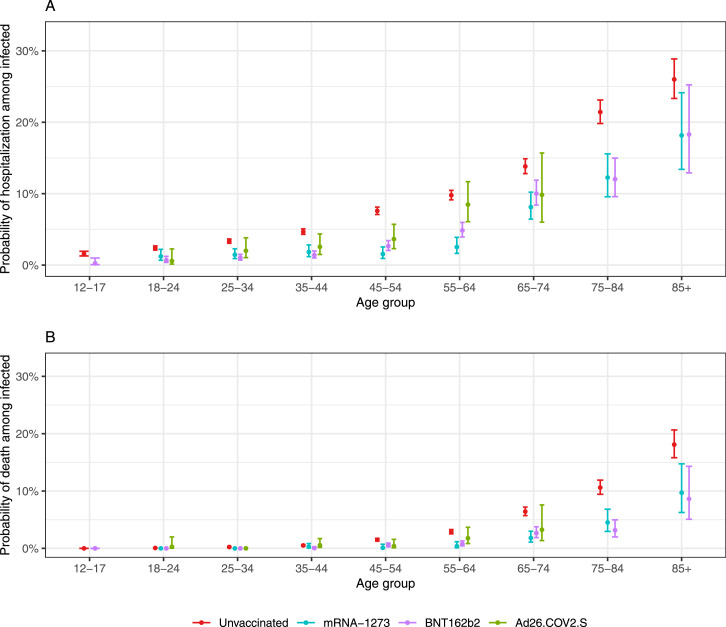
Estimated probability of adverse outcomes from COVID-19 among individuals with a laboratory-confirmed SARS-CoV-2 infection with 95% confidence intervals. Two vaccinated groups are compared to unvaccinated with the different vaccination manufacturers denoted with color. For Ad26.COV2.S, the estimates and confidence intervals for the 75–84 and 85+ are not shown due to small sizes. (A) Hospitalizations; (B) Deaths.

We fit a statistical model to determine if the vaccines provided further protection against COVID-19 hospitalization and death among SARS-CoV-2 infected individuals. Specifically, we estimated the age-group-specific risk of COVID-19 hospitalization and death conditioned on individuals testing positive for SARS-CoV-2 as a function of time since becoming fully vaccinated. Due to small sample sizes we did not run the analysis for Ad26.COV2.S, and for deaths, we combined the mRNA-1273 and BNT162b2data and only considered age groups above 65 years. We did not find clear evidence of waning for these conditional risks (see ***Supplementary Figures* S3** and **S4**). We therefore, fit a version of the model with constant conditional risk for each age group, which provided enough statistical power to obtain an estimate for all age groups and vaccine manufacturers. The probability for COVID-19 hospitalization after SARS-CoV-2 infection for those that were vaccinated was at least twice lower for all age groups except the 85 and older for which the probability was over 1.5 times lower. Similarly, the probability of COVID-19 death after SARS-CoV-2 infection was over three times lower for all age groups, except the 85 and older for whom it was about twice as low (Figure 4; ***Supplementary Table* S4**). We did not find statistically significant evidence that the Ad26.COV2.S vaccine provided added protection against COVID-19 hospitalization for people 75 and older, but the sample size was small (***Supplementary Table* S4**).

### COVID-19 hospitalizations and deaths avoided

We calculated, for each age group, the expected number of COVID-19 hospitalizations and deaths that would have been observed had the rates in each vaccinated group been equal to the rates of the unvaccinated group. We computed this for each vaccine manufacturer (***Supplementary Figure* S5**). In total, for mRNA-1273, BNT162b2, and Ad26.COV2.S, we saw a difference in COVID-19 hospitalizations of 2,599; 3,044; and 307, respectively, between what was expected with the unvaccinated rates and what was observed. For deaths, the observed difference was 954, 1,057, and 103, respectively. The grand total difference shows at least 5,950, COVID-19 hospitalizations and 2,106, COVID-19 deaths were avoided.

### No evidence of confounding

This an observational study and that, although we controlled for age, gender, and incidence rate, it is possible that there were unknown and unobserved confounders that were not accounted for, such as difference in behavior. Therefore, to help address these concerns, we examined vaccine effectiveness during the days right after the first dose. If the vaccinated and unvaccinated groups are comparable, apart from the vaccine effect, we expect to see no difference in infection rates (effectiveness = 0), since during the first days, the vaccine has not yet taken effect. The data are consistent with the two groups being comparable in terms of risk behaviors (***Supplementary Figure* S6**). Note that we observed effectiveness estimates near 1 (instead of 0) right after the first dose was administered, however, this could be expected because SARS-CoV-2 infected individuals were discouraged from getting vaccine until they recovered. This implies that on the day of the first dose we should see effectiveness close to 1. As the days go by, effectiveness drops to around 0 as expected, before it starts increasing, consistent with the individuals starting to acquire immunity.

## Discussion

Our analyses demonstrate that all vaccines were effective at reducing risks of SARS-CoV-2 infection, COVID-19 hospitalization, and death across all age groups. This was evident in mid July when a preliminary analysis of these data^14^ led the government to impose a series of vaccine mandates between July 22 and August 19, 2021. Daily vaccination rates increased after this and by September 27, 2021, Puerto Rico passed Massachusetts, Vermont, Maine, and Connecticut to become the US jurisdiction with the highest vaccination rate. The results of this current report are currently being used to develop recommendations regarding boosters shots. For example, we found that the protection provided by the mRNA-1273 and BNT162b2 vaccines were stronger and longer lasting than Ad26.COV2.S and, as a result, the Puerto Rico Department of Health is recommending mRNA-1273 and BNT162b2 boosters for individuals that received their Ad26.COV2.S over two months ago. We also found that the effectiveness of all vaccines decreased over time, implying that, to reach herd immunity, boosters shots are needed. These results also raise the question of weather new vaccine mandates should require booster shots after a certain time period of receiving the final dose. Although we removed individuals infected within the last 3 months to define the susceptible unvaccinated population, we note that because not all infected individuals are detected, the population might be slightly smaller leading to underestimates of the vaccine effectiveness. This implies that the vaccine may be even more effective than described here.

It is important to note that this is an observational study and not a randomized clinical trial. Although we controlled for confounding factors that were coded and available for analyses, it is possible that there were unknown and unobserved confounders that were not accounted for, such as chronic health conditions and immunocompromising conditions, and behavioral changes after vaccination. Note also that due to difference in testing behavior between vaccinated and unvaccinated it is possible that infections in these two groups may result in differences in severity. However, the findings described here agree with general trends observed in the clinical trials and our sensitivity analysis exploring effects due to confounding did not reveal concerning results.^2^^,34^

It is also important to note that the statistical analysis presented here is completely reproducible. Furthermore, the Puerto Rico Department of Health has developed a reproducible pipeline that can update the dataset needed to perform the analysis. We can therefore update the results regularly to continue providing guidance on public health decisions in Puerto Rico, and beyond.

## Contributors

RAI conceived this study. ICG was the project administrator of the vaccination process in Puerto Rico. EGN coordinated and managed the Department of Health databases. EGN and MMRF curated the data under RAI's supervision. MMRF and RAI performed the statistical analysis and wrote the first draft of the manuscript. All authors gave feedback on the manuscript for important intellectual content. All authors gave final approval of the version to be published. All authors had final responsibility for the decision to submit for publication.

## Data sharing

The data and code needed to reproduce this analysis is available here: https://github.com/rafalab/vax-eff-pr. The data wrangling code for curation is also available. The raw data sits on Department of Health HIPAA compliant databases and is not publicly available as it includes personal identifiers. However, the code for the matching algorithm is also available at https://github.com/rafalab/vax-eff-pr.

## Declaration of interests

ICG was a vaccine preventable disease speaker for Merck from 2006 to 2018. ICG was a meningococcal disease speaker for Pfizer from 2016 to 2019. ICG received support from Merck in the form of meals for lecture presentations. All other authors declare no competing interests.

## Appendix Supplementary materials

Image, application 1

Image, application 2

Image, application 3

Image, application 4

Image, application 5

Image, application 6

Image, application 7

Image, application 8

Image, application 9

Image, application 10

Image, application 11

Image, application 12

Image, application 13

Image, application 14

Image, application 15

Image, application 16
