# Supplementary material for: Effectiveness estimates of three COVID-19 vaccines based on observational data from Puerto Rico
Source: Lancet Reg Health Am. 2022 Feb 24;9:100212. doi: 10.1016/j.lana.2022.100212 (PMC8867062; doi:10.1016/j.lana.2022.100212)
Supplement: Supplementary file 1 [file mmc1.docx]

***Supplementary Figure Legends***

**Figure S1:** Cumulative number of fully vaccinated individuals by age group and vaccine manufacturer. Each pane represents a vaccine manufacturer and colors represent the different age groups.

**Figure S2:** Number of individuals for each possible value of days since fully vaccinated. We show values for before and after the Delta variant became dominant (solid versus dashed) and vaccine manufacturer (colors).

**Figure S3:** Estimates for risk of COVID-19 hospitalization among individuals with laboratory-confirmed SARS-CoV-2 infections as a function of days since being fully vaccinated. The 99% point-wise estimates are included. Each pane shows a different age group and vaccine types are denoted by color. The Ad26.COV2.S was not included due to small sample sizes.

**Figure S4:** Estimates for risk of COVID-19 death among individuals with laboratory-confirmed SARS-CoV-2 infections as a function of days since being fully vaccinated. The 99% point-wise estimates are included. Each pane shows a different age group. The Ad26.COV2.S was not included due to small sample sizes.

**Figure S5:** Reduction in risk provided by the different vaccines for individuals across age groups. The graph shows estimated expected outcomes based on estimated unvaccinated rates and those observed. **(A)** COVID-19 hospitalizations; **(B)** COVID-19 deaths.

**Figure S6:** We computed vaccine effectiveness, defined as 1 minus observed laboratory-confirmed SARS-CoV-2 infections divided by what is expected without vaccination, for each day since being fully vaccinated. (A) Data for the mRNA-1273 and BNT162b2 were combined, and results are shown for each age group. (B) Data for Ad26.COV2.S with age groups combined to provide statistical power.
