# Supplementary material for: Effectiveness estimates of three COVID-19 vaccines based on observational data from Puerto Rico
Source: Lancet Reg Health Am. 2022 Feb 24;9:100212. doi: 10.1016/j.lana.2022.100212 (PMC8867062; doi:10.1016/j.lana.2022.100212)
Supplement: Supplementary file 2 [file mmc2.docx]

## Supplementary Methods

### Statistical analyses

We applied models that accounted for age, gender, and, since many more tests were performed on weekdays than weekends, day of the week. We used the estimates obtained from fitting these models to quantify effectiveness against a SARS-CoV-2 infection and relative risks for COVID-19 hospitalizations and deaths. The models used for the different analyses are described below.

#### Time-varying effectiveness against a laboratory-confirmed SARS-CoV-2 infection by age group and vaccine manufacturer

 We defined $Y_{t,a,g}^{(u)}$ as the number of laboratory-confirmed SARS-CoV-2 infections observed on day $t$ for non-vaccinated individuals of gender $g$ (male or female) and age group $a$ (12-17, 18-24, 25-34, 35-44, 45-54, 55-64, 65-74, 75-84, or 85+). We assumed these counts followed a Poisson distribution with rate $N_{t,a,g}^{(u)}\mu_{a,g}\left( t \right)$ with $N_{t,a,g}^{(u)}$ the population size of the unvaccinated susceptible population in Puerto Rico on day $t$ from demographic group defined by $g$ and $a$ and $\mu_{a,g}\left( t \right)$ defined by

$$\text{log}\left\{ \mu_{a,g}\left( t \right) \right\}=\alpha_{i}+f_{a}\left( t \right)+\sum_{w=1}^{7} \gamma_{w}1_{w}\left( t \right)\text{ with }\sum_{g=1}^{2} \alpha_{g}=0\text{ and }\sum_{w=1}^{7} \gamma_{w}=0 (1)$$

with $\alpha_{g}$ a gender effect, $f_{a}(t)$ a cubic-spline representing an age-specific trend that accounts for the change in incidence across time, and $1_{w}(t)=1$ indicator functions for each day of the week $w\in\{\text{Sunday},\text{Monday},\ldots\text{Friday}\}$. This last term is used to account for the fact that the number of tests varies across days of the week (more tests performed on Mondays and less on Sundays, for example).

To obtain $N_{t,a,g}^{(u)}$we first computed an estimate of the population size of the demographic group, defined by $a$ and $g$, in Puerto Rico. Then for each day $t$ we subtract the number of individuals receiving their first dose and any individual infected in the previous 3 months.

We defined $Y_{t,d,a,g}^{(v)}$ as the number of laboratory confirmed SARS-CoV-2 infections observed on day $t$ for individuals of gender $g$ and age group $a$, that were fully vaccinated with vaccine $v$ (mRNA-1273, BNT162b2, or Ad26.COV2.S), $d$ days previous to the positive test. We assumed these counts followed an Poisson distribution with expected rate defined by

$$\text{E}\left( Y_{t,d,a,g}^{(v)} \right)=N_{t,d,a,g}^{(v)} \mu_{a,g}(t) \text{exp}\left\{ \beta_{a}^{(v)}(d) \right\}. (2)$$

Here $N_{t,d,a,g}^{(v)}$ is the population size on day $t$ of individuals of gender $g$, age group $a$ that were fully vaccinated with vaccine $v$, $d$ days before day $t$, $\mu_{a,g}(t)$ is the incidence rate defined for model (1) on date $t$ among the non-vaccinated individuals of gender $g$ and age $a$, and $\beta_{a}^{(v)}(d)$ is the age-specific log relative risk of vaccine $v$, $d$ days after the first dose was administered. Note that if we assume $\beta_{a}^{(v)}(d)$ is a cubic-spline, then the model defined by (1) and (2) is a Generalized Linear Model (GLM) and we can we find the Maximum Likelihood Estimate for $\beta_{a}^{(v)}(d)$ using Iteratively Reweighted Least Square, as implemented by the *glm* function in R. We can use standard GLM methodology, also implemented by the *glm* function, to estimate standard errors and confidence intervals. The use of the cubic splines permitted us to obtain precise estimates of the effects even when the some of the counts were small as they borrowed statistical power across the time scales.

The transformation $f(x)=1-\text{exp}(x)$ can be used to convert the $\beta$ (log relative risks) to effectiveness.

Note that we can use this model for the hospitalization and deaths outcomes by simply redefining the counts $Y_{t,d,a,g}^{(v)}$ based on counts of these outcomes.

#### COVID-19 hospitalization and death relative risk conditioned on a laboratory confirmed SARS-CoV-2 infection

Note: in this section, to avoid introducing more mathematical symbols, we repurpose the $Y,N,\alpha,\beta$ and $\gamma$ notation and, because we fit a model separately to each age group, we do not use the $a$ index.

To estimate the further protection provided by the vaccine in reducing hospitalization and deaths among infected individuals we examined the proportion of cases that had hospitalizations or deaths. To do this we defined $N_{i,d}$ as the number of laboratory-confirmed SARS-CoV-2 infections on day $d$ for group $i$. Groups were defined by gender (male or female) and vaccination status (unvaccinated, mRNA-1273, BNT162b2. or Ad26.COV2.S). We defined $Y_{i,d}$ as the number of these that had the hospitalization or death outcome. We then assumed that $Y_{i,d}$ followed a binomial distribution with $N_{i,t}$ trials and success probability $p_{i,d}$ defined by:

$$\text{log}\frac{p_{i,d}}{1-p_{i,d}}=\alpha_{g}+\beta_{i}^{(v)}(d)\text{ with }\sum_{g=1}^{2} \alpha_{g}=0$$

with $\alpha_{g}$ a sex effect and $\beta_{a}^{(v)}(d)$ the time-varying conditional risk vaccination status $v$. We fit this model using the Iteratively Reweighted Least Square algorithm. In our analysis we first fit the model without the $\alpha_{g}$ and exclude the Ad26.COV2.S vaccine data due to small sizes obtained after stratifying by age group, and, for deaths, we combined the mRNA-1273, or BNT162b2 data. After not finding strong evidence in favor of the need for time-varying effect, we fit a version of the model with $\beta_{a}^{(v)}(d)$ a constant with respect to $d$ to each vaccine group, but this time including the sex effect and for all three vaccines.
