# Supplementary material for: Effectiveness estimates of three COVID-19 vaccines based on observational data from Puerto Rico
Source: Lancet Reg Health Am. 2022 Feb 24;9:100212. doi: 10.1016/j.lana.2022.100212 (PMC8867062; doi:10.1016/j.lana.2022.100212)
Supplement: Supplementary file 3 [file mmc3.docx]

**Supplementary Tables**

**Table S1:** Chronology of the COVID-19 vaccine rollout in Puerto Rico.

| Date | Event |
| --- | --- |
| Dec 12, 2020 | BNT162b2 COVID-19 vaccine (Pfizer-BioNTech) receives Emergency Use Authorization (EUA). |
| Dec 15, 2020 | The COVID-19 vaccine rollout begins with phase 1A which included healthcare workers, communities in long term care facilities and intellectual disabilities care facilities. |
| Dec 18, 2020 | mRNA-1273 COVID-19 vaccine (Moderna) receives EUA. |
| Jan 11, 2021 | Phase 1B commences with the vaccination of adults 65 and older. |
| Feb 2, 2021 | Puerto Rico's Secretary of Health signs administrative order establishing that for the next 28 days, vaccination will be exclusive for those 65 and older. |
| Mar 3, 2021 | Ad26.COV2.S COVID-19 vaccine (Johnson & Johnson) receives EUA. |
| Mar 11, 2021 | Secretary of Health signs administrative order establishing that for the following 30 days, first doses are to be administered exclusively to adults 60 and older with certain chronic conditions. |
| Mar 17, 2021 | Secretary of Health signs administrative order establishing that for the following 30 days, first doses are to be administered exclusively to adults 60 and older and 50 to 59 year olds with chronic conditions. |
| Mar 17, 2021 | Secretary of Health signs executive order authorizing the vaccination of personnel in food industry, drug companies, medical equipment, the public transport sector, air transport and maritime cargo. |
| Mar 29, 2020 | Phase 1C begins with the vaccination of people 50 and older and 35 and older with chronic conditions. |
| Apr 12, 2021 | Phase 3 begins with vaccination available to everyone 16 and older. |
| May 12, 2021 | CDC recommends vaccination for people 12 years and older. |
| Jul 22, 2021 | Secretary of Health signs administrative order stating that students 12 years and older must be vaccinated with at least one dose for in-person class in schools and universities. Similarly, school and university employees and contractors must be fully vaccinated. |
| Jul 28, 2021 | Governor signs executive order establishing that all public agencies of the Executive Branch shall require employees who work in-person to receive a COVID-19 vaccine. |
| Jul 29, 2021 | Secretary of Health signs administrative order stating that 1) every public employee who works in person or remotely must be fully vaccinated by September 30, 2021, and 2) that every contractor who works with the Puerto Rico Department of Health must be fully vaccinated by September 30, 2021. |
| Aug 5, 2021 | Governor signs executive order requiring contractors and their employees who work in-person or frequently visit their offices, 2) all employees of the healthcare facilities, 3) all employees of hotels, paradores, and lodgings, and 3) all guests of hotels, paradores, lodgings, and short-term rentals to receive a COVID-19 vaccine. |
| Aug 9, 2021 | Secretary of Health signs administrative order stating that school and university employees and contractors must be fully vaccinated. However, if they fall within the religious or health exemptions category, they must present a weekly negative SARS-CoV-2 test. |
| Aug 11, 2021 | Governor signs executive order stating that any establishment-either indoor or outdoor-that sells prepared food or drink (eg. restaurants and chinchorros), must require all employees who work there in person to be fully vaccinated with a COVID-19 vaccine and that from August 23rd 2021, food businesses have two options 1) 100% capacity if business visitors present COVID-19 vaccination card, negative antigen or molecular test or a positive antigen or molecular test and a certificate of recovery for visitors who have had an infection, or 2) 50% capacity if business does not require any evidence from visitors. |
| Aug 19, 2021 | Governor signs executive order establishing that beauty salons, barber shops, aesthetics salons, spas, gyms, childcare centers, grocery stores, convenience stores, casinos, and gas stations must require and ensure that all employees who work there in person to be fully vaccinated with a COVID-19 vaccine. |

**Table S2:** Number of individuals, by age group, receiving the first or dose or being fully vaccinated before and after June 15, 2021 when Delta variant became dominant in Puerto Rico.

| Manufacturer | Age group | First dose before June 15 | First dose after June 15 | Fully vaccinated before June 15 | Fully vaccinated after June 15 |
| --- | --- | --- | --- | --- | --- |
| mRNA-1273 | 12-17 | - | - | - | - |
| mRNA-1273 | 18-24 | 83,939 | 15,292 | 57,902 | 29,674 |
| mRNA-1273 | 25-34 | 110,483 | 19,999 | 79,378 | 35,570 |
| mRNA-1273 | 35-44 | 119,665 | 14,959 | 91,126 | 30,368 |
| mRNA-1273 | 45-54 | 134,791 | 13,612 | 105,874 | 28,979 |
| mRNA-1273 | 55-64 | 141,241 | 12,019 | 113,709 | 25,767 |
| mRNA-1273 | 65-74 | 151,762 | 7,758 | 132,276 | 13,784 |
| mRNA-1273 | 75-84 | 92,267 | 4,428 | 79,441 | 7,750 |
| mRNA-1273 | 85+ | 32,192 | 1,662 | 26,568 | 2,807 |
| BNT162b2 | 12-17 | 127,236 | 76,138 | 31,055 | 148,770 |
| BNT162b2 | 18-24 | 99,418 | 42,560 | 75,669 | 48,395 |
| BNT162b2 | 25-34 | 132,231 | 56,473 | 107,017 | 58,280 |
| BNT162b2 | 35-44 | 149,665 | 46,583 | 124,579 | 51,410 |
| BNT162b2 | 45-54 | 169,118 | 37,772 | 146,481 | 41,622 |
| BNT162b2 | 55-64 | 179,512 | 31,752 | 159,083 | 33,588 |
| BNT162b2 | 65-74 | 140,172 | 22,509 | 128,225 | 19,404 |
| BNT162b2 | 75-84 | 82,878 | 13,566 | 75,945 | 10,813 |
| BNT162b2 | 85+ | 24,529 | 5,275 | 22,067 | 3,890 |
| Ad26.COV2.S | 12-17 | - | - | - | - |
| Ad26.COV2.S | 18-24 | 9,067 | 2,831 | 8,471 | 3,297 |
| Ad26.COV2.S | 25-34 | 13,237 | 5,594 | 12,032 | 6,559 |
| Ad26.COV2.S | 35-44 | 17,894 | 5,405 | 16,527 | 6,458 |
| Ad26.COV2.S | 45-54 | 22,187 | 4,839 | 20,721 | 5,822 |
| Ad26.COV2.S | 55-64 | 23,781 | 3,858 | 22,511 | 4,597 |
| Ad26.COV2.S | 65-74 | 10,084 | 2,197 | 9,531 | 2,563 |
| Ad26.COV2.S | 75-84 | 6,015 | 1,084 | 5,759 | 1,260 |
| Ad26.COV2.S | 85+ | 2,676 | 409 | 2,590 | 481 |

**Table S3:** Person years, SARS-CoV-2 infections (cases), hospitalizations, and deaths from December 29, 2020, the first day that individuals were fully vaccinated, to October 15, 2021 by vaccination group.

|  | Person years | Cases | Hospitalizations | Deaths |
| --- | --- | --- | --- | --- |
| Total | 1,990,288 | 82,089 | 4,718 | 1,237 |
| Unvaccinated | 1,053,120 | 60,321 | 3,933 | 1,055 |
| mRNA-1273 | 371,307 | 6,430 | 244 | 62 |
| BNT162b2 | 510,526 | 12,927 | 423 | 87 |
| Ad26.COV2.S | 55,335 | 2,411 | 118 | 33 |

**Table S4:** Estimated probability of hospitalizations and deaths from COVID-19 among individuals infected with SARS-CoV-2. Note that the mRNA-1273 vaccine and Ad26.COV2.S vaccine are not approved for individuals younger than 18. Thus, no probabilities are reported for the 12-17 age group for this vaccine.

| Outcome | Age group | Unvaccinated | BNT162b2 | mRNA-1273 | Ad26.COV2.S |
| --- | --- | --- | --- | --- | --- |
| Hospitalization | 12-17 | 0.02 (0.01,0.02) | 0.00 (0.00,0.01) | - | - |
| Hospitalization | 18-24 | 0.02 (0.02,0.03) | 0.01 (0.00,0.01) | 0.01 (0.01,0.02) | 0.01 (0.00,0.02) |
| Hospitalization | 25-34 | 0.03 (0.03,0.04) | 0.01 (0.01,0.02) | 0.01 (0.01,0.02) | 0.02 (0.01,0.04) |
| Hospitalization | 35-44 | 0.05 (0.04,0.05) | 0.01 (0.01,0.02) | 0.02 (0.01,0.03) | 0.03 (0.01,0.04) |
| Hospitalization | 45-54 | 0.08 (0.07,0.08) | 0.03 (0.02,0.03) | 0.02 (0.01,0.03) | 0.04 (0.02,0.06) |
| Hospitalization | 55-64 | 0.10 (0.09,0.10) | 0.05 (0.04,0.06) | 0.03 (0.02,0.04) | 0.08 (0.06,0.12) |
| Hospitalization | 65-74 | 0.14 (0.13,0.15) | 0.10 (0.08,0.12) | 0.08 (0.06,0.10) | 0.10 (0.06,0.16) |
| Hospitalization | 75-84 | 0.21 (0.20,0.23) | 0.12 (0.10,0.15) | 0.12 (0.10,0.16) | 0.25 (0.16,0.37) |
| Hospitalization | 85+ | 0.26 (0.23,0.29) | 0.18 (0.13,0.25) | 0.18 (0.13,0.24) | 0.46 (0.29,0.65) |
| Deaths | 12-17 | 0.00 (0.00,0.00) | 0.00 (0.00,0.00) | - | - |
| Deaths | 18-24 | 0.00 (0.00,0.00) | 0.00 (0.00,0.00) | 0.00 (0.00,0.00) | 0.00 (0.00,0.02) |
| Deaths | 25-34 | 0.00 (0.00,0.00) | 0.00 (0.00,0.00) | 0.00 (0.00,0.00) | 0.00 (0.00,0.00) |
| Deaths | 35-44 | 0.01 (0.00,0.01) | 0.00 (0.00,0.00) | 0.00 (0.00,0.01) | 0.01 (0.00,0.02) |
| Deaths | 45-54 | 0.02 (0.01,0.02) | 0.01 (0.00,0.01) | 0.00 (0.00,0.01) | 0.00 (0.00,0.02) |
| Deaths | 55-64 | 0.03 (0.03,0.03) | 0.01 (0.00,0.01) | 0.00 (0.00,0.01) | 0.02 (0.01,0.04) |
| Deaths | 65-74 | 0.06 (0.06,0.07) | 0.03 (0.02,0.04) | 0.02 (0.01,0.03) | 0.03 (0.01,0.08) |
| Deaths | 75-84 | 0.11 (0.09,0.12) | 0.03 (0.02,0.05) | 0.05 (0.03,0.07) | 0.09 (0.04,0.19) |
| Deaths | 85+ | 0.18 (0.16,0.21) | 0.09 (0.05,0.14) | 0.10 (0.06,0.15) | 0.36 (0.20,0.55) |

**Table S5:** Estimated risk of hospitalizations from COVID-19 among individuals infected with SARS-CoV-2. Note that the mRNA-1273 vaccine and Ad26.COV2.S vaccine are not approved for individuals younger than 18. Thus, no risk estimates are reported for the 12-17 age group for these vaccines.

| Age group | BNT162b2 | mRNA-1273 | Ad26.COV2.S |
| --- | --- | --- | --- |
| 12-17 | 6.34 (2.01, 38.51) | - | - |
| 18-24 | 3.49 (2.01, 6.79) | 1.94 (1.12, 3.78) | 4.14 (1.33, 24.97) |
| 25-34 | 3.24 (2.23, 4.94) | 2.31 (1.49, 3.85) | 1.67 (0.92, 3.49) |
| 35-44 | 3.33 (2.38, 4.82) | 2.56 (1.68, 4.13) | 1.83 (1.10, 3.34) |
| 45-54 | 2.86 (2.20, 3.79) | 4.91 (3.06, 8.57) | 2.08 (1.35, 3.45) |
| 55-64 | 2.01 (1.62, 2.54) | 3.87 (2.55, 6.23) | 1.15 (0.83, 1.67) |
| 65-74 | 1.38 (1.13, 1.69) | 1.70 (1.33, 2.21) | 1.40 (0.87, 2.45) |
| 75-84 | 1.78 (1.39, 2.31) | 1.75 (1.34, 2.32) | 0.85 (0.53, 1.45) |
| 85+ | 1.42 (0.98, 2.15) | 1.43 (1.03, 2.05) | 0.56 (0.33, 1.06) |

**Table S6:** Estimated risk of death from COVID-19 among individuals infected with SARS-CoV-2. Note that the mRNA-1273 vaccine and Ad26.COV2.S vaccine are not approved for individuals younger than 18. Thus, no risk estimates are reported for the 12-17 age group for these vaccines In addition, age groups 18 to 44 are excluded because the death count is close to 0 and the estimated risk not defined.

| Age group | BNT162b2 | mRNA-1273 | Ad26.COV2.S |
| --- | --- | --- | --- |
| 45-54 | 14.64 (3.29, 257.46) | 2.91 (1.65, 5.69) | 3.79 (1.21, 22.92) |
| 55-64 | 7.64 (2.92, 30.89) | 3.84 (2.29, 7.08) | 1.63 (0.83, 3.82) |
| 65-74 | 3.52 (2.17, 6.20) | 2.41 (1.69, 3.56) | 1.97 (0.91, 5.53) |
| 75-84 | 2.34 (1.54, 3.77) | 3.34 (2.13, 5.58) | 1.14 (0.55, 2.89) |
| 85+ | 1.87 (1.20, 3.10) | 2.10 (1.25, 3.87) | 0.51 (0.28, 1.07) |
