# Supplementary figures and images for: Effectiveness estimates of three COVID-19 vaccines based on observational data from Puerto Rico
Source: Lancet Reg Health Am. 2022 Feb 24;9:100212. doi: 10.1016/j.lana.2022.100212 (PMC8867062; doi:10.1016/j.lana.2022.100212)

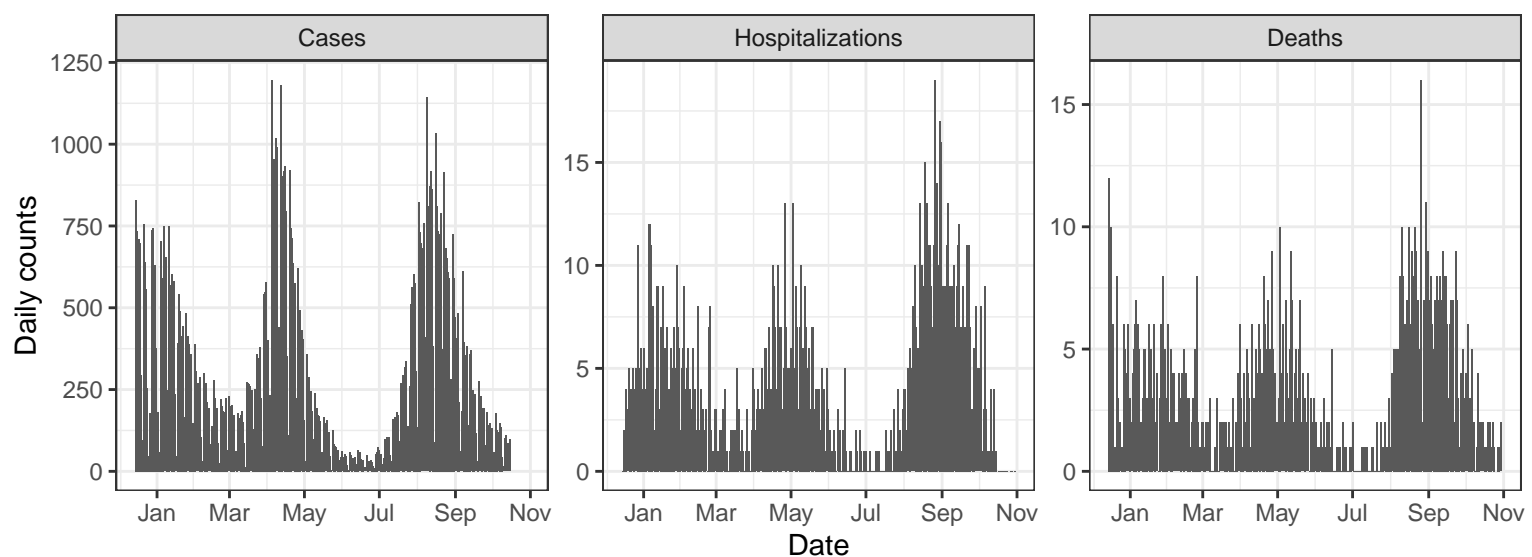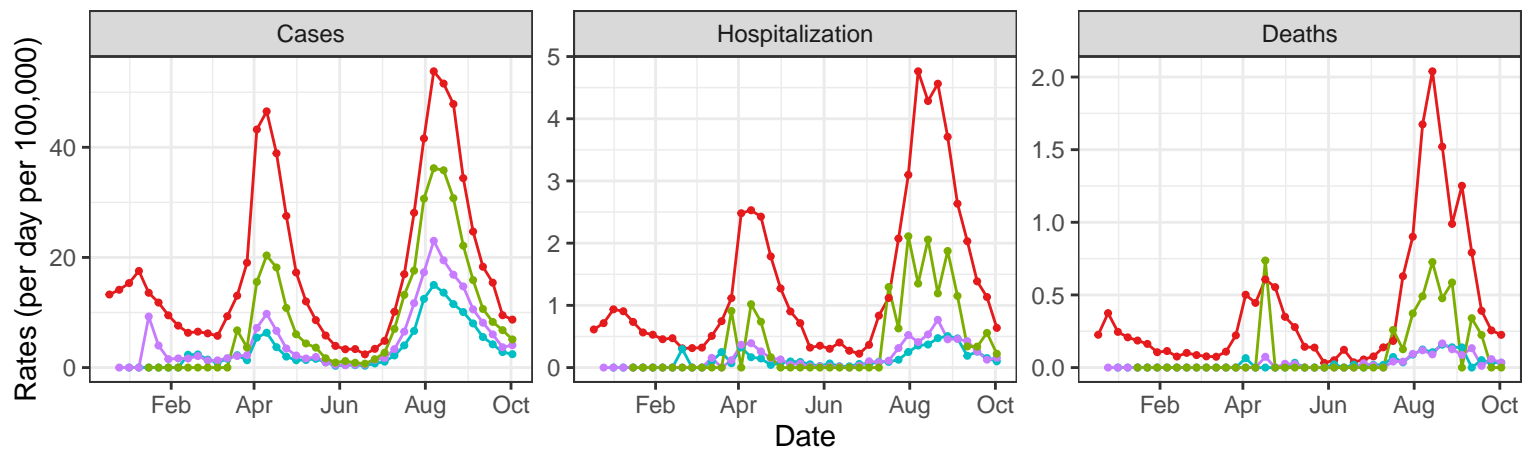

Vaccination status — Unvaccinated — mRNA-1273 — BNT162b2 — Ad26.COV2.S

Supplement: Supplementary file 4 [file mmc4.pdf]

B

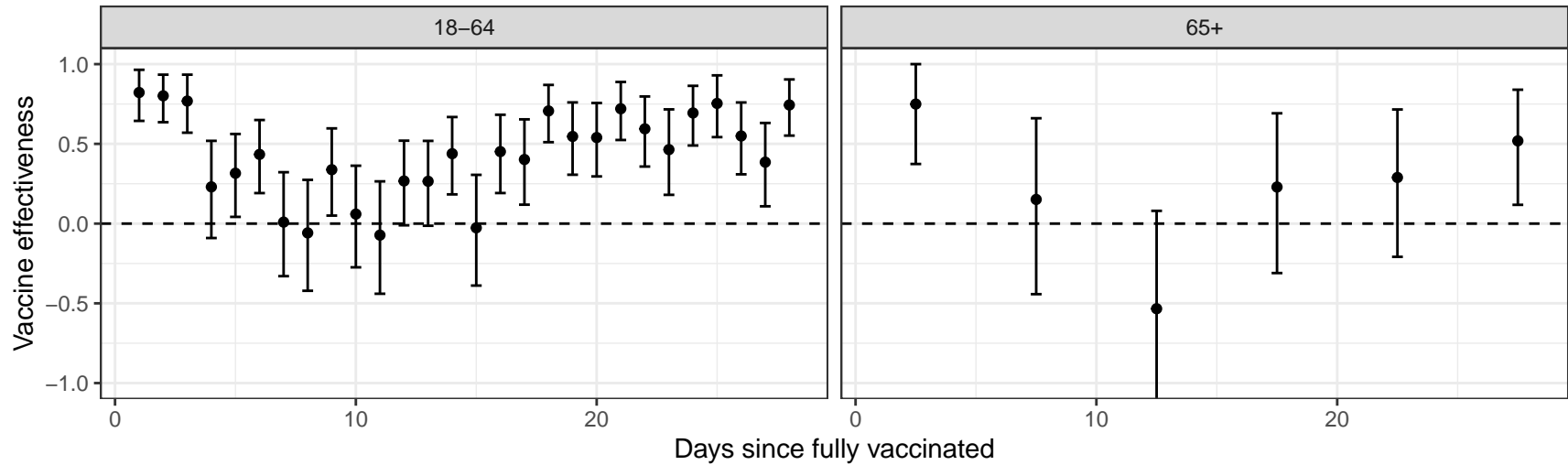

Supplement: Supplementary file 5 [file mmc5.pdf]

A

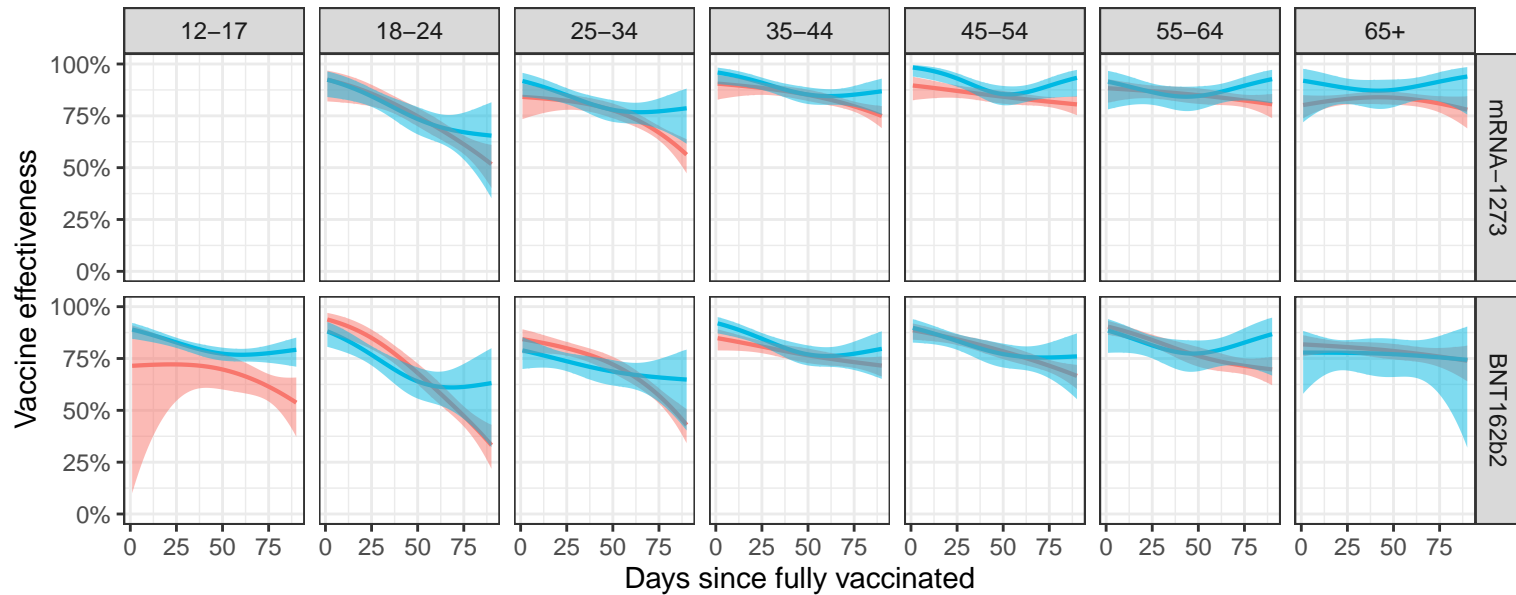

— Before June 15, 2021 — After June 15, 2021

Supplement: Supplementary file 6 [file mmc6.pdf]

A

COVID-19 hospitalizations

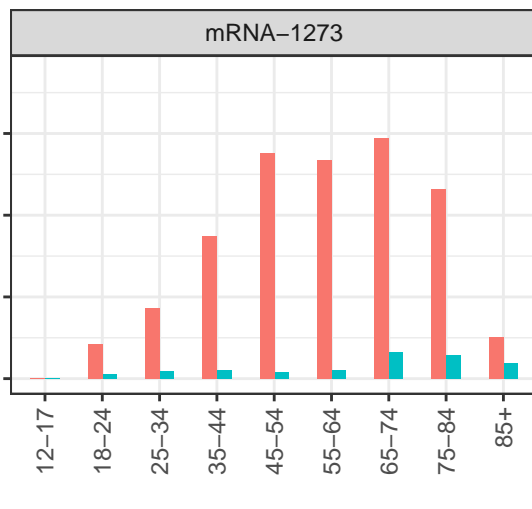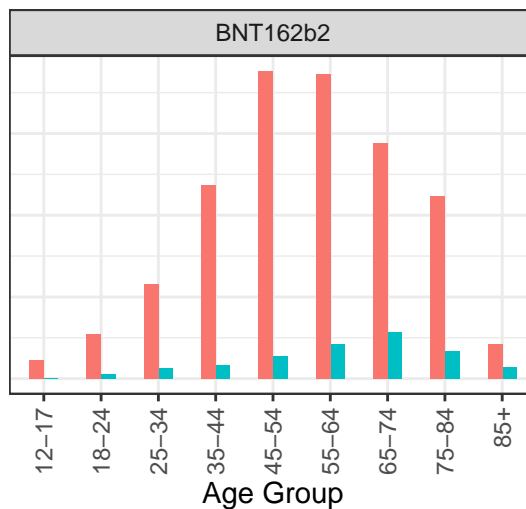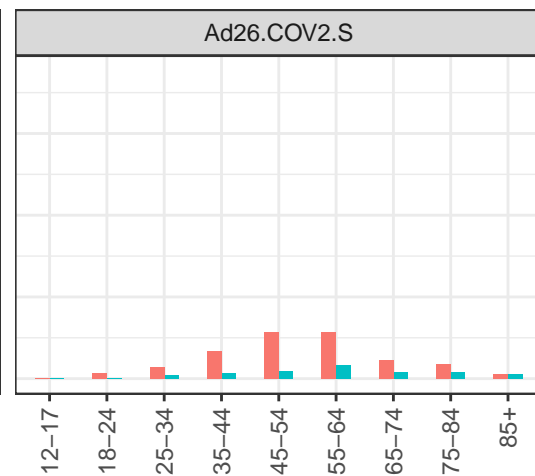

B

COVID-19 deaths

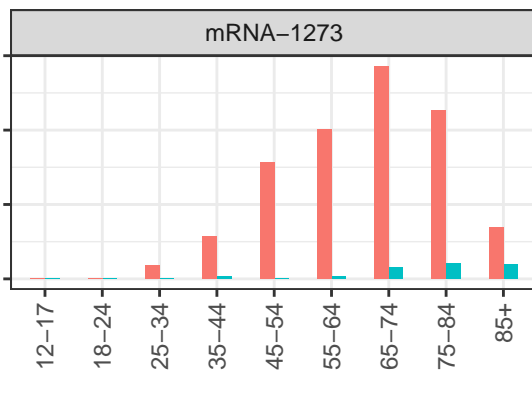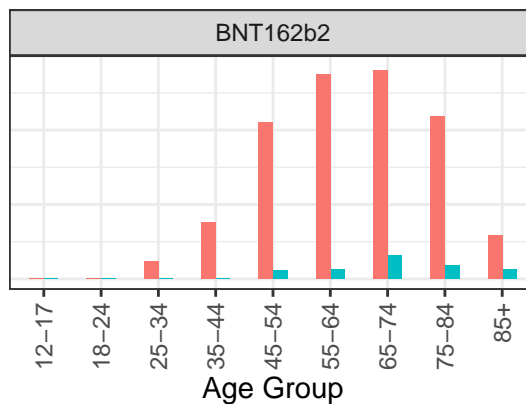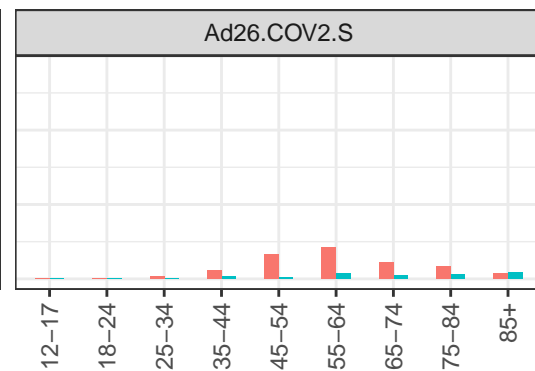

Expected Observed

Supplement: Supplementary file 7 [file mmc7.pdf]

B

Probability of death among infected

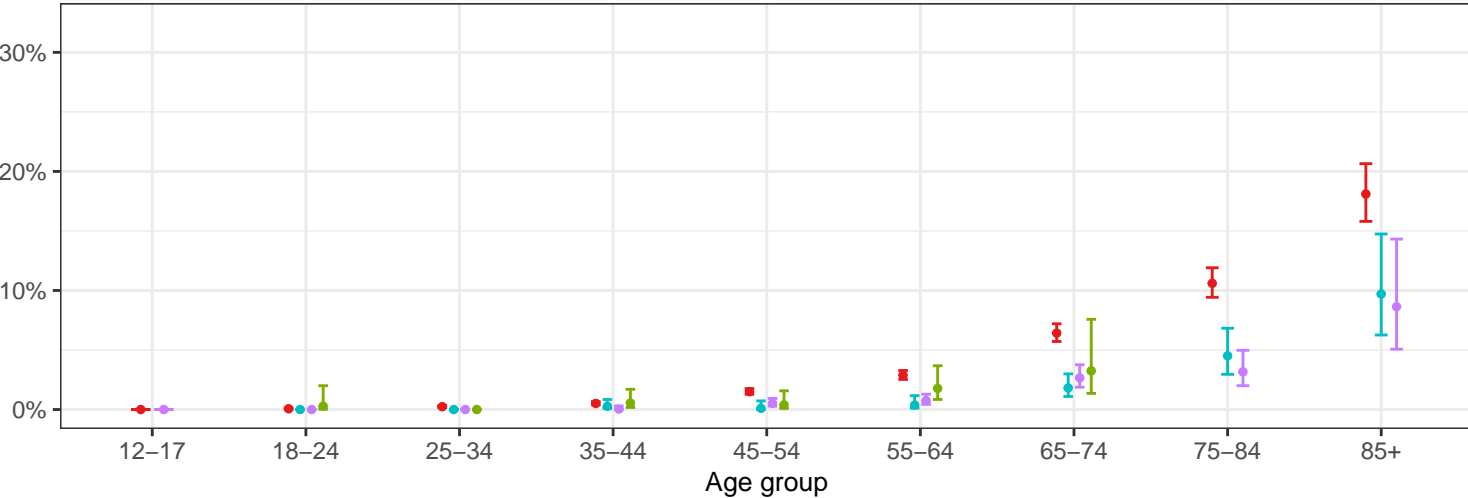

Unvaccinated mRNA-1273 BNT162b2 Ad26.COV2.S

Supplement: Supplementary file 8 [file mmc8.pdf]

A

Probability of hospitalization among infected

12-17

18-24

25-34

35-44

45-54

55-64

65-74

75-84

85+

Age group

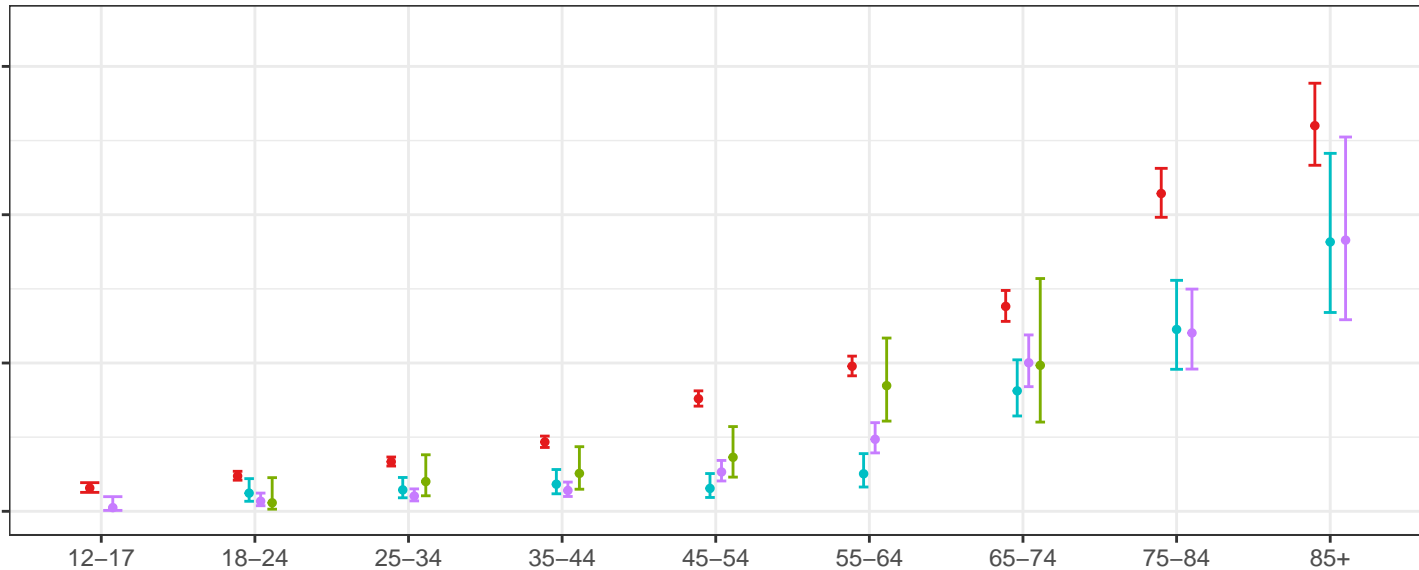

Supplement: Supplementary file 9 [file mmc9.pdf]

Fully vaccinated individuals

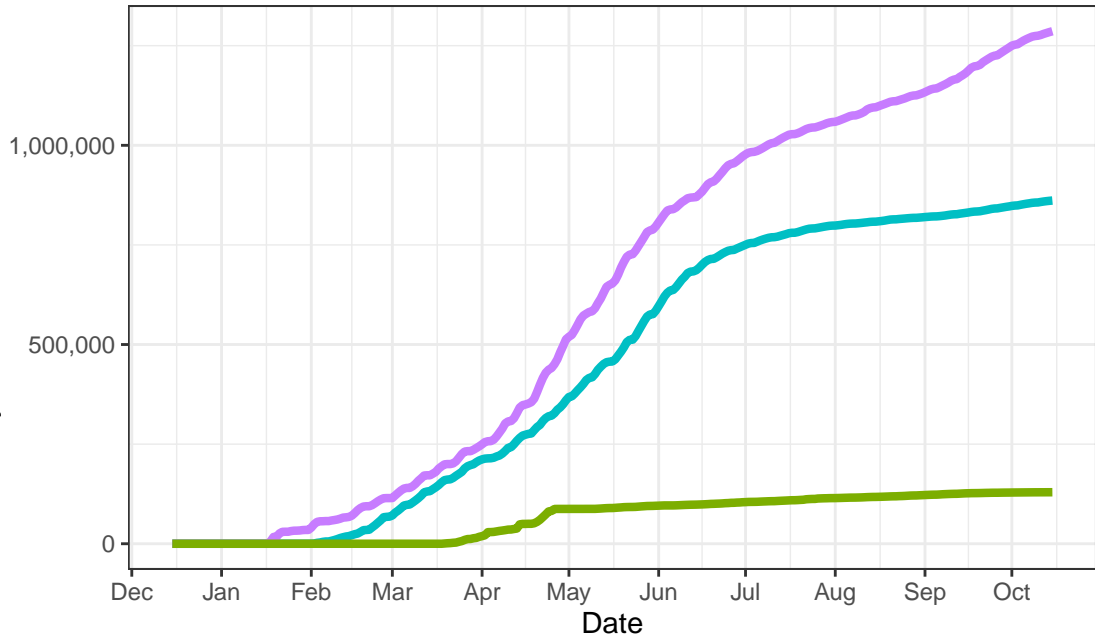

Vaccine   mRNA-1273   BNT162b2   Ad26.COV2.S

Supplement: Supplementary file 10 [file mmc10.pdf]

A

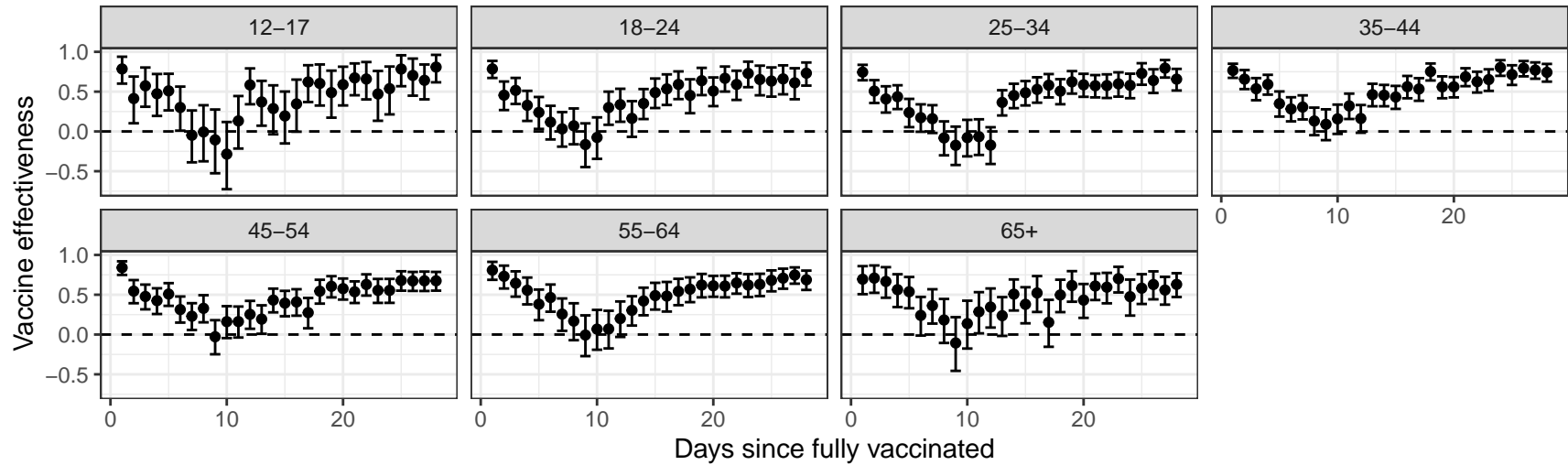

Supplement: Supplementary file 12 [file mmc12.pdf]

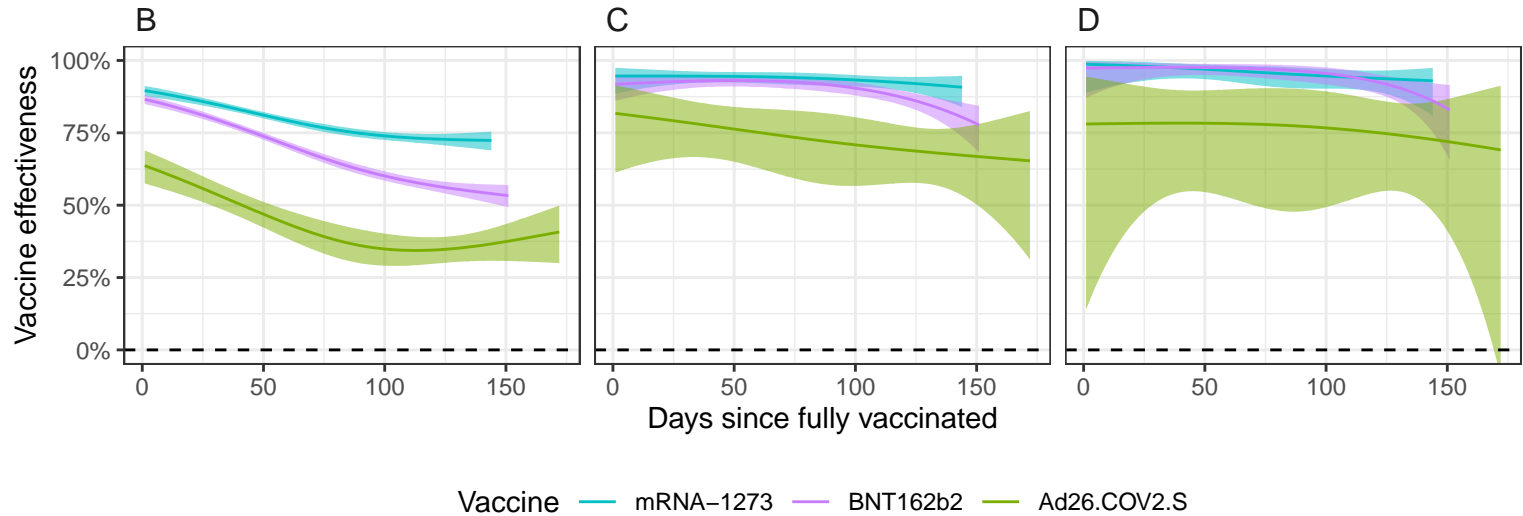

Supplement: Supplementary file 13 [file mmc13.pdf]

Risk of hospitalization given infection

65–74

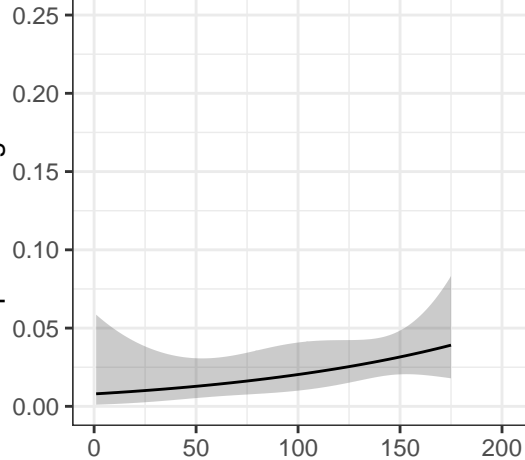

75–84

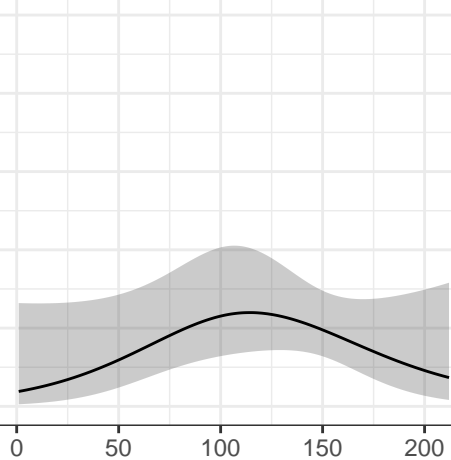

85+

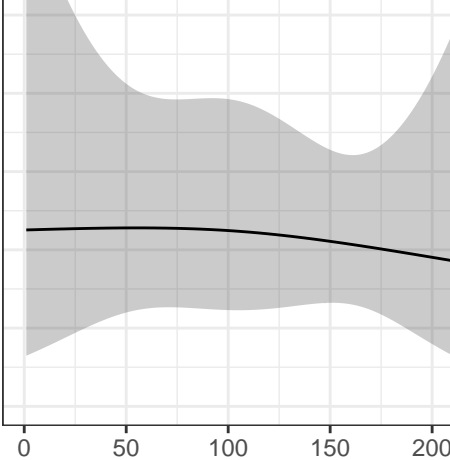

Days since fully vaccinated

Supplement: Supplementary file 14 [file mmc14.pdf]

Risk of hospitalization  
given infection

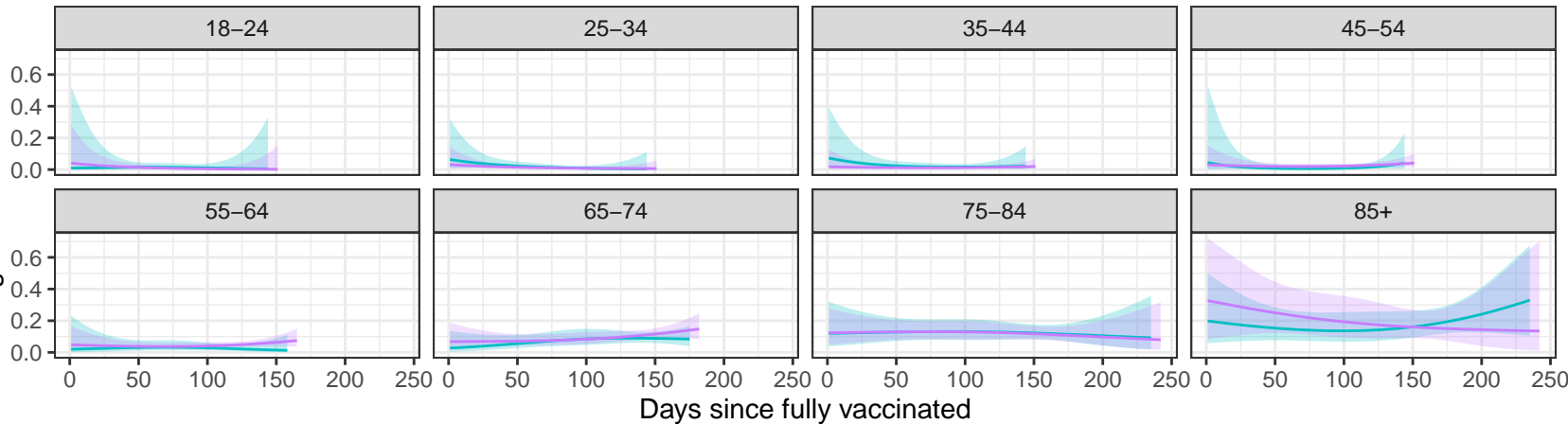

Vaccine mRNA-1273 BNT162b2

Supplement: Supplementary file 15 [file mmc15.pdf]

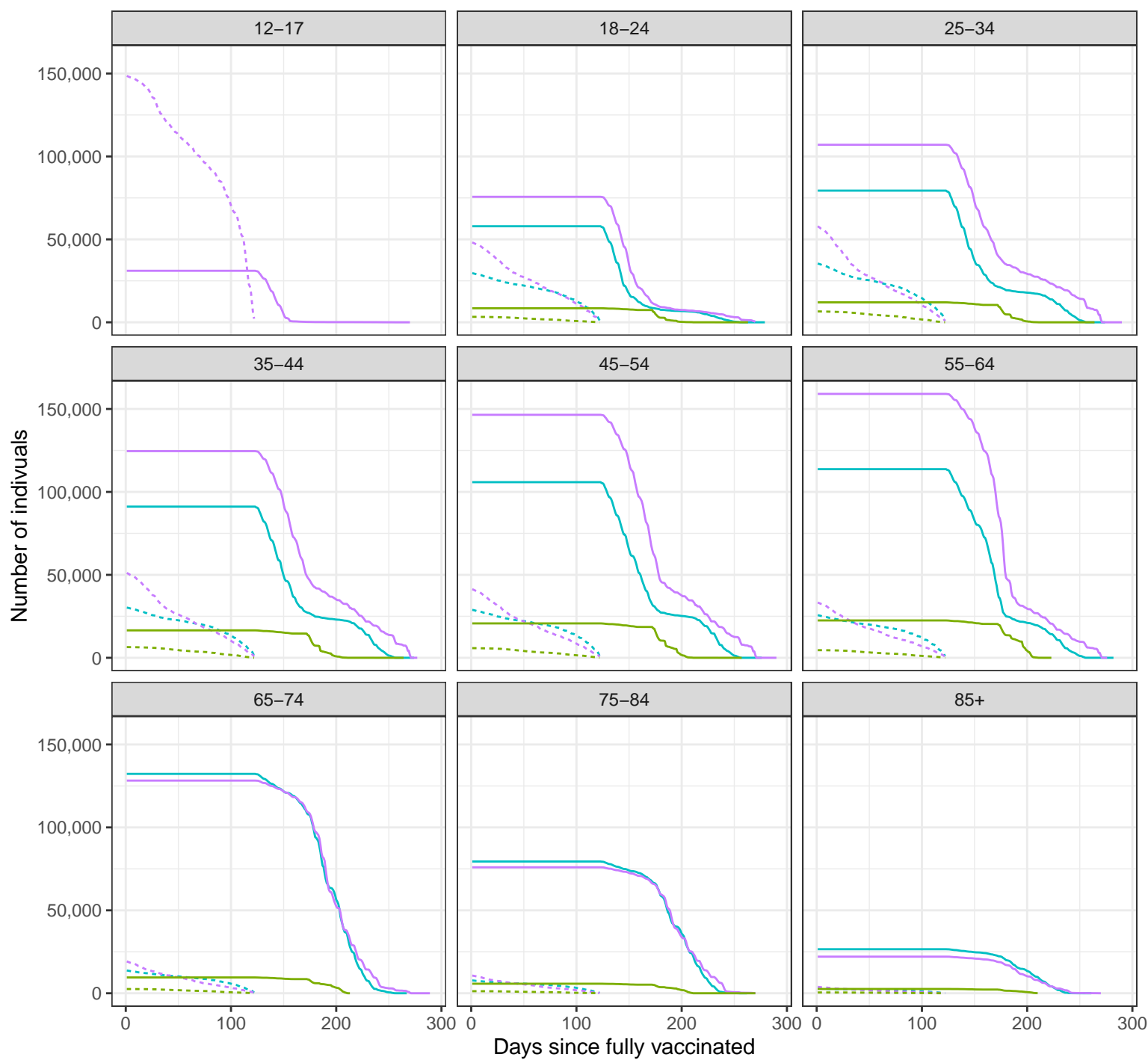

Vaccine mRNA-1273 BNT162b2 Ad26.COV2.S Before June 15, 2021 After June 15, 2021

Supplement: Supplementary file 16 [file mmc16.pdf]
