# Supplementary material for: Effectiveness estimates of three COVID-19 vaccines based on observational data from Puerto Rico
Source: Lancet Reg Health Am. 2022 Feb 24;9:100212. doi: 10.1016/j.lana.2022.100212 (PMC8867062; doi:10.1016/j.lana.2022.100212)
Supplement: Supplementary file 11 [file mmc11.pdf]

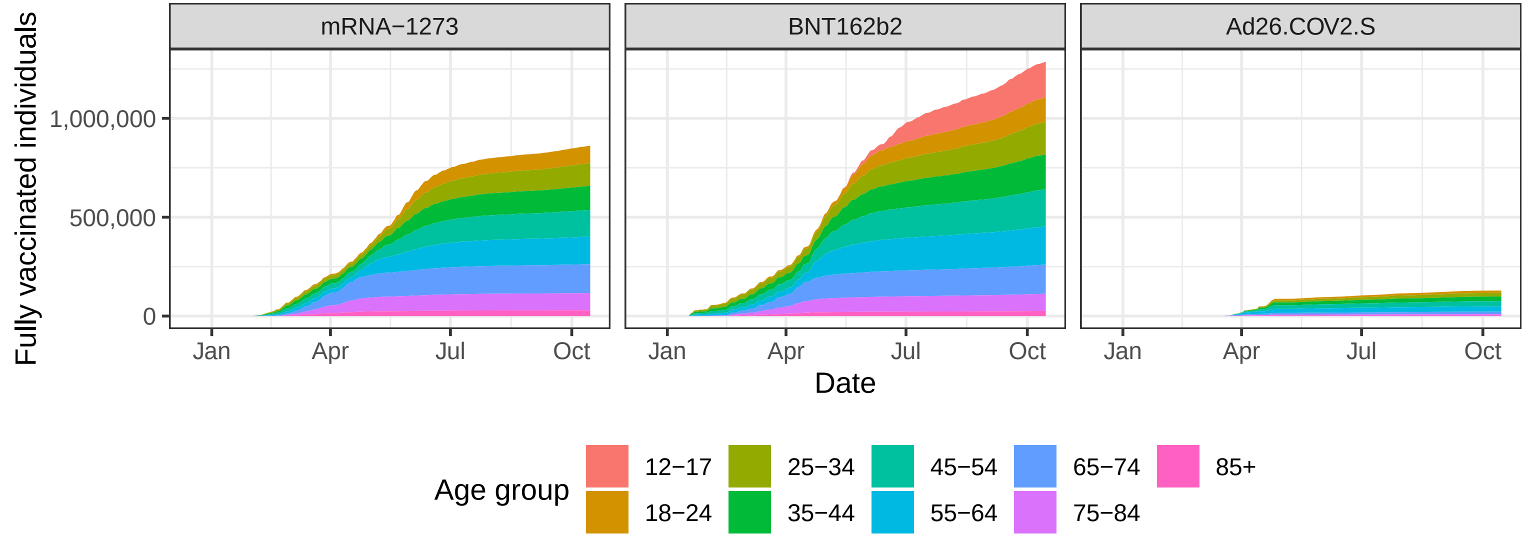
mRNA−1273
BNT162b2
Ad26.COV2.S
Fully vaccinated individuals
1,000,000
500,000
0
Jan
Apr
Jul
Oct
Date
Age group
12−17
18−24
25−34
35−44
45−54
55−64
65−74
75−84
85+
